# CRISPR as agent: a metaphor that rhetorically inhibits the prospects for responsible research

**DOI:** 10.1186/s40504-018-0088-8

**Published:** 2018-11-13

**Authors:** Leah Ceccarelli

**Affiliations:** Department of Communication, University of Washington, Box 353740, Seattle, WA 98195-3740 USA

**Keywords:** Asilomar conference on recombinant DNA, Berg letter, CRISPR research moratorium, International summit on human gene editing, Jennifer Doudna

## Abstract

In 2015, a group of 18 scientists and bioethicists published an editorial in *Science* calling for “open discourse on the use of CRISPR-Cas9 technology to manipulate the human genome” and recommending that steps be taken to strongly discourage “any attempts at germline genome modification” in humans with this powerful new technology. Press reports compared the essay to a letter written by Paul Berg and 10 other scientists in 1974, also published in *Science*, calling for a voluntary deferral of certain types of recombinant DNA experimentation. A rhetorical analysis of the metaphors in these two documents, and in the summary statements that came out of the respective National Academy of Sciences conferences they instigated, shows that while they have a lot in common, they are different in at least one important way. The more recent texts deploy conceptual metaphors that portray the biotechnology in question as an autonomous agent, subtly suggesting an inevitability to its development, in contrast to the earlier texts, which portray the scientists who are using the technology as the primary agents who take action. Rhetorical moves depicting biotechnology as an agent in the 2015 texts hint at contemporary skepticism about whether humans can restrain the forward momentum of science and technology in a global context, thus inhibiting scientists from imagining a consequential role for themselves in shaping the future of responsible research.

## Introduction

*Slate* begins its article on “the fuzzy regulations surrounding DIY synthetic biology” with a link to a citizen scientist’s “CRISPR kit” available online for $150 (Olmstead 2017). In its synthetic biology "cheat-sheet,” *Slate* identifies CRISPR as part of the lingo that one must know, right along with the term “synthetic biology” itself and “BioBricks” (Brogan 2017). *The New Yorker* proclaims in breathless prose that we might soon transcend Darwinian evolution as we are “propelled by CRISPR and other tools of synthetic biology” to “alter the genetic destiny" of our species (Specter 2017). Meanwhile, *Wired* worries about the “synthetic bioweapons” that state enemies could create “with new gene editing tools like Crispr-Cas9” (Niler 2017). Rightly or wrongly, and for good or ill, CRISPR is coming to be seen as a technology closely associated with synthetic biology.

*Science* magazine named CRISPR its “Breakthrough of the Year” in 2015, calling it a “molecular marvel.” With a metaphor that compares it to a person, the journal says that CRISPR “was conceived” in 2007, got a “birth announcement” in 2012, took its “first steps” in 2013, experienced “a massive growth spurt” in 2014, then quickly “matured,” so that by the end of 2015, it “shows its power” with its “mind-blowing” capabilities. The journal concludes its tribute with a claim that “we all now live in CRISPR’s world” (Travis 2015). It is this idea, that CRISPR-Cas9 gene editing technology is a living thing with its own authority and ability and that we now live in *its* world, that I will examine in this paper. When we metaphorically envision CRISPR as an agent with the power to alter the human germline, rather than as an agency that we make a conscious decision to use or not use, we are carried along by a linguistic and cognitive momentum that makes it less likely that we will orient toward this technology as a tool under the control of scientists and science regulators who make decisions about whether or not particular synthetic biology projects should be undertaken. To make this case about the influence of our language choices on our efforts to engage in responsible research and innovation, I will conduct a metaphor analysis of two similar texts calling for research pauses for regulatory deliberation, each written in a different era. One was written about CRISPR and the other was written about an earlier genetic engineering technology known as recombinant DNA.

Both texts were published in *Science*. Not long before that journal named CRISPR the most significant scientific discovery of 2015, a group of 18 scientists and bioethicists published an editorial there calling for “open discourse on the use of CRISPR-Cas9 technology to manipulate the human genome.” In the editorial, the authors “strongly discourage, even in those countries with lax jurisdictions where it might be permitted, any attempts at germline genome modification for clinical application in humans, while societal, environmental, and ethical implications of such activity are discussed among scientific and governmental organizations. … This will enable pathways to responsible uses of this technology, if any, to be identified” (Baltimore et al. 2015a, 36-7). The corresponding author of the editorial was Jennifer Doudna, one of the co-inventors of CRISPR-Cas9 technology.

The fact that one of the scientists responsible for the CRISPR breakthrough was an author of the plea that we stop to think about “a prudent path” for responsible uses of it was a newsworthy event. *The New York Times* reported that this call “for a worldwide moratorium” on use of a new method of genetic engineering was not without precedent, since the essay was similar to a call made by scientists in the 1970s for a moratorium on recombinant DNA research (Wade 2015). Other science journalists made the same comparison, calling forth the memory of that earlier text that had made a similar entreaty (Duncan 2015; Vogel 2015). The analogy was facilitated by the fact that two scientists, Paul Berg and David Baltimore, were the first two authors on both texts.

The earlier call for a moratorium to discuss options for promoting responsible research with a new technology for genetic manipulation was published as a letter in *Science* in 1974, signed by 11 scientists. The first author, Berg, was one of the inventors of recombinant DNA technology. He and his co-authors promised to stop doing certain types of recombinant DNA experiments themselves, and they used the moral force of their example to ask “scientists throughout the world” to join with them in “voluntarily deferring” that kind of research until its “potential hazards” have been better evaluated or until adequate methods are developed for managing those hazards (Berg et al. 1974).

Although the two texts are about different genetic engineering technologies and are separated by more than 40 years, those who mention them in the same breath are right in saying that they are similar. Both the Doudna essay and the Berg letter begin with a description of the new biotechnology in question, and both end with a set of four recommendations for addressing the risks associated with that new technology. The last item on both lists is a proposal for an international meeting to discuss the next steps to be taken; and both texts were successful in bringing about such meetings. In response to the Doudna essay, the National Academy of Sciences co-sponsored an International Summit on Human Gene Editing in December 2015 that was parallel to the Asilomar conference that they had held in May 1975 in response to the Berg letter.

In a previously published essay, I performed a rhetorical analysis of these two texts to see if there were significant differences between these two calls for responsible research coming from two different eras (Ceccarelli 2018). I focused in particular on sentence structure, to see where responsibility was situated in subject-predicate pairings throughout the texts. I found that while the two texts were similar in purpose, they differed in style. In the 2015 text, the technology in question is often situated as the subject of the sentence, making it an autonomous agent of change. This sentence structure suggests an inevitability to the development of bioscience, evoking a technological determinism in which CRISPR acts, and we humans are forced to live in its world. In contrast, the 1974 text uses subject-predicate pairings that more often portray the scientists who are using the biotechnology as the subject of sentences. In 1974, it is the scientists’ world, and whether or not genetic engineering technologies are used is a decision that they make.

In that earlier rhetorical analysis, I also looked briefly at the summary statement that came out of the 2015 International Summit on Human Gene Editing, and I found the same thing that I had found in the Doudna essay that called it forth. The statement includes sentences that situate CRISPR as the main subject with active voice constructions that portray it as an agent of change, while using passive voice constructions to portray scientists as powerless. I concluded from that analysis that recent rhetorical choices that depict a scientific technique as an autonomous agent of change could reflect contemporary skepticism about the capability of human agents to restrain the forward momentum of science and technology in a global context, thus undercutting the sincerity of the more recent attempts to ensure responsible research. In this follow-up study, I take that argument one step further by looking at the metaphors that appear in these two texts and in the conference statements that followed each of them.

## Method and theory

My approach to metaphor analysis in this paper draws from rhetorical theorist Kenneth Burke’s understanding of language as a “terministic screen.” Burke’s evocative metaphor to describe the operation of language tools (such as metaphor) encourages us to understand that the words we choose “necessarily constitute a corresponding kind of screen” or filter on our perceptions (Burke 1966, 50). His “dramatistic” approach to language turns our attention to “the necessarily *suasive* nature of even the most unemotional scientific nomenclatures,” helping us to see that “even if any given terminology is a *reflection* of reality, by its very nature as a terminology it must be a *selection* of reality; and to this extent it must function also as a *deflection* of reality” (Burke 1966, 45).

Burke’s perspective toward language aligns with the approach taken by George Lakoff and Mark Johnson in their highly influential *Metaphors We Live By* (Lakoff and Johnson 1980). Focusing on conceptual metaphors, that is, on the conventionalized or dormant metaphors that underlie all speech, Lakoff and Johnson help us to recognize the limitations of a text’s implicit worldview, and urge us to consider what alternatives might be suppressed by what appear to be otherwise routine language choices. For example, when Lakoff and Johnson point to the cluster of war-terms that contemporary Americans use to talk about argument (he *attacked* my claim, I *defended* my point, she *shot down* my *position*), they help us to see how conceptual metaphors of aggression guide our thinking about argumentation, and this allows us to consider alternatives that are currently deflected by our metaphoric imagination (such as “argument as dance”). Identifying implicit conceptual metaphors is a powerful way of uncovering the worldview of the authors of a text.

In addition to thinking about conceptual metaphors as terministic screens, another way that I will draw from Burke’s rhetorical theory to perceive what is going on in the texts that I analyze in this paper is by considering his dramatistic pentad of key terms: agent (that is, subject or character), act (what is done), scene (setting or background), agency (prop or tool), and purpose. Identifying the ratio of one key term to another in a text, we can see what is selected by the implicit worldview of that artifact and what is simultaneously deflected from our attention (Burke 1945). Given what I found in my earlier study, I expect the Doudna essay to include metaphors that treat the biotechnology of CRISPR (which is literally a tool, or agency) as an agent in its own right that is responsible for the acts that follow its entry onto the scene of modern science, thus privileging an agency/act ratio. I expect the Berg letter to use language that portrays scientists as agents who are responsible for how they choose to use, or not use, the agency of recombinant DNA technology, thus privileging an agent/act ratio. The implications of these choices are significant. The agency/act ratio serves as a sign of our contemporary lack of confidence in our ability to positively influence a runaway technology, and reinforces that belief by closing off our capacity to imagine scientists effectively undertaking responsible research and innovation. An agent/act ratio imagines scientists and science regulators as capable of controlling the biotechnological agencies that they use, and encourages them to take responsibility for the acts that are carried out when they use those agencies.

In addition to drawing from Burke, I draw from metaphor theorists who encourage scholars of rhetoric to identify the tenor and vehicle, or target domain and source domain, of any particular metaphor (Richards 1936). This is an important methodological move to avoid conflating metaphoric language that is being used to talk about different things. For instance, consider a study published in *The American Journal of Bioethics* that analyzed all the metaphors associated with CRISPR in key American newspapers and popular science publications over the previous two years (O’Keefe et al. 2015). In a response to that article, my colleagues and I used metaphor theory to identify the tenors and vehicles of the principal metaphors listed there. We did so to distinguish between those metaphoric vehicles that are generally associated with the tenor of the genome, and those metaphoric vehicles that are specifically used for the tenor of CRISPR or, alternatively, for what CRISPR is used to do (Nelson et al. 2015). In particular, we found that metaphors that refer to code or blueprint are about the genome upon which CRISPR works. The metaphor of molecular scalpel refers to CRISPR itself, as a tool. Metaphors of editing or targeting refer to what CRISPR is used to do, but not to CRISPR itself. The tenors for CRISPR that are implied by the use of editing or targeting vehicles are most likely a word processor and high-tech weaponry, respectively (that is, things that are typically used for editing and targeting). Keeping tenors and vehicles straight helps a scholar of rhetoric avoid conflating vehicles that have been introduced for different tenors, so that we do not end up comparing apples to oranges.

So for example, the authors of that otherwise insightful metaphor analysis proposed “ecological metaphors” as a positive alternative to “editing” or “targeting” metaphors for CRISPR (O’Keefe et al. 2015, 8). This sounds like a good idea, until you realize that “ecological” is not a verb, like editing and targeting are; it is an adjective. With a metaphoric vehicle coming from the source domain of ecology (i.e., an ecological metaphor), we could envision the genome in its environment as part of an ecosystem, which is an image that has much to recommend it. But we would then have to imagine CRISPR not as “ecological” per se, but as a technology for *altering* that ecosystem, such as geoengineering, which has a much less positive connotation for readers than the term “ecological” does. Without a clear distinction between metaphoric vehicles that are being applied to the tenors of the biotechnology itself, the thing upon which the biotechnology is being used, and the action taken with the biotechnology, the implications of any recommendation regarding metaphors become muddled.

So in the analysis I perform in this paper, I am going to keep these things distinct by identifying the different tenors that the metaphoric vehicles are being used to talk about, namely, the scene, the agency, or the act, which in this case is the genome, CRISPR, and what CRISPR is used to do, or more broadly, the genes, the genetic technology, and what that genetic technology is used to do. Those tenors are, of course, three parts of Burke’s pentad – the other two being agent and purpose, or in this case, the scientist and why the scientist uses, or chooses not to use, the technology. My method of rhetorical reading will reconstruct the conceptual metaphors that are implicit in the texts, while distinguishing tenor and vehicle to make explicit the often unnoticed terministic screens that reflect, select, and deflect our perception of biotechnology and our responsible use of it.

## The Berg letter

The first sentence of the 1974 Berg letter serves as the exception to the rule that is followed in the rest of that text. It presents the biotechnology being considered as a metaphoric agent. (To identify the metaphors in my analysis below, I will use italics to highlight the out-of-place terms.) “Recent advances in techniques for the isolation and rejoining of segments of DNA now *permit construction* of biologically active recombinant DNA molecules in vitro” (Berg et al. 1974). This first sentence compares the tenor of “advances in techniques” (recombinant DNA technologies) to the vehicle of metaphoric government officials with the authority to “permit” or license some unnamed others (presumably scientists) to undertake the “construction” of a biological product. This metaphor personifies biotechnology by treating it as an autonomous agent. However, it is a heterodox metaphor in this text because it turns out that it is the only one in the entire letter that does this.

In the rest of the letter, biotechnology is represented in the more standard way, as something used by scientists, not as an independent agent that magnanimously uses its authority to grant power to others. Consider, for example, the first sentence of the second paragraph of the Berg letter, which begins: “Several groups of scientists are now planning to use this technology.” At the end of the Berg letter, the authors say “our concern for the possible unfortunate consequences of indiscriminate applications of these techniques motivates us” to act (Berg et al. 1974). There are no metaphoric reversals in these sentences. Scientists are the agents and technology is an agency or tool that is being used, or potentially misused, by those scientists after they contemplate their options and then choose whether or not to act responsibly.

Terms suggesting the metaphoric vehicle of a construction worker or artisan do reappear in this text, but they are attached most often to the tenor of scientists, not recombinant DNA technology. The terms “construct,” “create,” and “link” or “join,” and variants thereof appear 13 times in the text, referring to acts that researchers have either performed or will perform with genetic material, or are choosing to voluntarily defer from performing. For example, DNA restriction endonucleases “have been used to *create* new types of biologically functional bacterial plasmids carrying antibiotic resistance markers,” and to “*link*” ribosomal DNA from frogs to DNA from a bacterial plasmid; and “several groups of scientists are now planning to use this technology to *create* recombinant DNAs.” When the writers of the letter call for “scientists throughout the world” to join them in voluntarily deferring certain kinds of experiments, they identify the actions to be avoided as the “*construction* of new, autonomously replicating bacterial plasmids that might result in the introduction of genetic determinants for antibiotic resistance” into bacterial strains that do not have such resistance, or the “*construction* of new bacterial plasmids containing” such resistance. “Plans to *link* fragments” should be carefully weighed, and since the “*joining* of any foreign DNA to a DNA replication system *creates* new recombinant DNA molecules whose biological properties cannot be predicted with certainty, such experiments should not be undertaken lightly” (Berg et al. 1974). These terms taken in isolation might seem literal, not metaphorical, but clustered together, they generate the image of the scientist as an artisan or blue collar worker manufacturing a product with commercial value. Researchers who are used to thinking of themselves as professionals doing intellectual labor are described with terministic screens that characterize them as agents who manufacture products, construction workers capable of building or choosing not to build something by withholding their labor.

## The Doudna essay

Significantly, none of these terms (construct, create, link, or join) appears in the Doudna essay, even though CRISPR is often attached in public discourse to synthetic biology, a field that is filled with metaphoric vehicles of construction, manufacturing, and creative work (O’Keefe et al. 2015; McLeod and Nerlich 2017). Related terms that might better fit the specific action of CRISPR-Cas9 technology, such as “fix” or “repair,” also fail to appear in the Doudna essay. The term “editing” appears twice, but in neither case is it attached to a scientist-agent who might be performing the act of editing, or refusing to do so. Instead, the term appears in the noun phrase “genome editing,” a process that some “cells and tissues have undergone” (Baltimore et al. 2015a, 37).

The term “engineering” appears frequently in the Doudna essay (18 times), but almost always as part of a noun phrase that identifies the technology being discussed: genome (or genomic, or germline) engineering. The term is not introduced as an action that researchers are taking; they are never said to be “engineering” something. Instead, the term is presented as the name of the technology itself, the method, or the field. And as a technology or method or field, it is often conceived as an agent in its own right.

This is the biggest difference between the texts from the two eras. As opposed to the Berg letter, the Doudna essay includes not just one, but several sentences that metaphorically imagine the biotechnology being discussed as an autonomous agent with almost unlimited power. The first sentence of the essay begins the personification of CRISPR technology: “Genome engineering technology *offers* unparalleled potential for modifying human and nonhuman genomes. In humans, it *holds* the promise of curing genetic disease, while in other organisms it *provides* methods to reshape the biosphere for the benefit of the environment and human societies” (Baltimore et al. 2015a, 36). CRISPR technology is thus conceived as an agent that not only offers, but holds and provides, and the power it has is almost godlike, to *reshape* the biosphere itself.

In the second paragraph of the Doudna essay, the “CRISPR-Cas9 system” is likewise seen as a powerful agent that “enables” and “allows.” Readers are told that “Advances in DNA sequencing capabilities … *have provided* critical information about the genetic changes that influence the development of disease.” Here, the technology is metaphorically imagined as not only a powerful agent, but an intelligent one too, offering not just methods but knowledge. At the end of this paragraph, readers are told that “a rapidly expanding *family* of CRISPR-Cas9-derived technologies is *revolutionizing* the fields of genetics and molecular biology” (Baltimore et al. 2015a, 36). It is significant that scientists are not leading the revolution here; instead, it is the technologies themselves that are revolutionizing the areas of research. The family metaphor is also telling. CRISPR Cas9-derived technologies are anthropomorphized through the vehicle of the family, imagined not only to be connected with each other in a kin relationship, but to be part of a “rapidly expanding” family, suggesting autonomous reproduction or growth of those technologies.

As with the Berg letter, there are exceptions to the rule. In the initial one sentence abstract of the Doudna essay, “CRISPR-Cas9 technology” is envisioned as a tool that one might “use” to achieve some goal (Baltimore et al. 2015a, 36). However, in most of the 2015 essay, CRISPR-Cas9 is imagined not as a tool to be used by scientists, but as a living agent, metaphorically conceived as a being that is subject to the same laws of evolution that guide every other living thing. It emerges, develops, and contains within itself certain potentials for action. For example, when considering a lesson from the earlier recombinant DNA era about the need for transparency and open discussion to gain public trust, the authors say “that lesson is amplified today with the *emergence* of CRISPR-Cas9 technology and the imminent prospects for genome engineering” (Baltimore et al. 2015a, 38). CRISPR-Cas9 technology is thus imagined to be something that is not invented, or even discovered, by scientists; instead, it emerges, and carries with it imminent prospects, as if it were a newly evolved species of organism with an ominous destiny. The discipline of genomic modification is also imagined to be alive through a subtle conceptual metaphor: “Given the speed with which the genome engineering field is *evolving* … there is an urgent need for open discussion of the merits and risks of human genome modification” (Baltimore et al. 2015a, 37). The whole technoscientific field is thus imagined to be developing with the vigor of a quickly evolving species.

To reinforce this vision, the term “develop” and its variants appears 11 times in this text, mostly referring to the technology itself having experienced “rapid development” (Baltimore et al. 2015a). Notably, the term “develop” only appears two times in the earlier Berg letter, and both times it has to do with the scientists’ prospective development of safety procedures, not with the rapid development of the technology itself (Berg et al. 1974). That is because biotechnology is most often treated as an agency or tool in the earlier text, where scientists laboring as construction workers or artisans are seen to be responsible for building things; in the later text, biotechnology is metaphorically conceived as a living agent with its own power to grow and evolve.

## The conference summary statements

The Asilomar Conference on Recombinant DNA Molecules that followed the Berg letter produced a summary statement of their discussion about “appropriate ways to deal with the potential biohazards” of research on recombinant DNA molecules (Berg et al. 1975, 991). The most significant outcome of that meeting was the identification of four levels of physical containment that scientists would adopt as a regulatory constraint over different kinds of recombinant DNA experimentation with different levels of risk. The belief that human beings have the power and responsibility to restrain the new biotechnology through the construction of physical containment is facilitated by the metaphoric imagination on display in the text. As in the Berg letter, the language of “construction” appears repeatedly in the Asilomar conference summary statement, with scientists described as doing work to construct “recombinant DNA molecules,” “special bacteria and vectors,” and a “new organism” at different points in the text (Berg et al. 1975). The terms *construct* and *create*, and variants thereof, appear nine times in the document.

We can see the scientist-as-agent worldview of the authors in the first paragraph of the summary statement, where they explain that “the use of recombinant DNA methodology promises to revolutionize the practice of molecular biology” (Berg et al. 1975, 991). Recall that in the Doudna essay, it was a rapidly expanding family of biotechnologies that was revolutionizing the field of molecular biology. The difference between the *use* of a technology promising to revolutionize a field and the technology *itself* revolutionizing that field might seem subtle, but it is a significant difference. In one case, it is scientists who are acting as responsible agents by using (or choosing not to use) the methodology; in the other it is the biotechnology that acts as the agent that is responsible for what follows.

This distinction might reflect differences in attitudes in the two eras about the power of humans to strategically act on a technology to influence it in a geopolitical context. Metaphorically, a Cold War rhetoric of containment is palpable in the Asilomar summary statement. The actual term “containment” appears an astounding 42 times in the text, along with four uses of the term “barrier” (Berg et al. 1975). The scientists at the 1975 meeting in Asilomar thought themselves capable of containing biohazardous vectors, hosts, and vector-host systems. By 2015, the rhetoric of containment seems less dominant in geopolitical discourse. So it should come as no surprise that the term never even appears in the statement coming out of the International Summit on Human Gene Editing. It might also come as no surprise that the latter statement offers no proposed solution to the risks and issues it identifies (Baltimore et al. 2015b).

The first two paragraphs of the 2015 International Summit on Human Gene Editing summary statement replicate some of the language patterns seen in the Doudna essay. Rather than saying that scientists produce biotechnology, the authors say that “scientific advances” (that is, technologies such as CRISPR) are what “have produced remarkable progress” and “have also raised important ethical and societal issues” (Baltimore et al. 2015b). It is as if the tools themselves, which in Burke’s pentad would normally be considered the agency, are the agents performing the act of moving science forward as well as causing problems in society. Rather than the people who are making the technologies being responsible for them, it is “fundamental research” that “has recently led to the *development* of powerful new techniques” (Baltimore et al. 2015b). The research itself (rather than the researcher) is the causal agent, and the technologies produced by that research are imagined as living things that are developing or growing. It is “these new techniques *that make it possible*” for gene editing to happen and “may also *enable* wide-ranging clinical applications in medicine” (Baltimore et al. 2015b). Again, we are to imagine new techniques as powerful agents who, as they grow and mature, make possible and enable other activities. The scientists who perform this research and invent the techniques are missing from these sentences and are thus deflected from our attention.

Toward the end of the statement, biotechnology is again metaphorically envisioned as a living thing with its own trajectory of change when we are told that “scientific knowledge *advances* and societal views *evolve*” (Baltimore et al. 2015b). It is not scientists who introduce new knowledge, or people who change societal views; instead, new knowledge and societal views seem to develop on their own, as if they are biological organisms following evolutionary laws.

## Conclusion

The difference in metaphoric worldviews embedded in the Berg letter and the Doudna essay are reflected in the stylistic choices made in the summary statements of the Asilomar conference and the International Summit on Human Gene Editing, respectively. In the two texts from the 1970s, the conceptual metaphors suggest an agent/act ratio, in which scientists are responsible for what they do with the technologies they use. As metaphoric construction workers, they envision and implement regulatory constraints that can help contain the risks of the products they build. In the texts from 2015, we see the technologies take on a life of their own, suggesting an agency/act ratio, in which the tools of synthetic biology are responsible for risky acts of alteration made on the human genome. Scientists are powerless in CRISPR’s world, carried along for the ride by a family of technologies that are revolutionizing biomedicine.

The consequences of these differing terministic screens can be significant. The rhetoric of the 2015 texts suggests a technological determinism in our current thinking that makes it hard for scientists to conceive of an active role for themselves in fostering responsible research and innovation. A fatalistic belief system has become part of our thinking about genetic engineering, “a dogma of ‘inevitability’ [that] encourages passivity and acquiescence. It discourages meaningful public involvement and inhibits scientists from considering the social implications of their research choices and from speaking out about their concerns for the uses and abuses of their work” (Weiner 2001, 217). This belief is reflected in the metaphors we select, and it simultaneously deflects alternative perspectives that would encourage responsible action by scientists and the introduction of regulatory constraints by policymakers.

It is true that in some ways, the deliberation occurring at the International Summit on Human Gene Editing was an improvement over what took place at the Asilomar conference over 40 years earlier. Unlike the earlier conference, which invited only scientists as participants, this one invited ethicists, historians, sociologists, and parent activists (Hogan 2016). It also included a more diverse and international group of participants (Jasanoff and Hurlbut 2018). But in another way, the outcome of the more recent meeting can be considered inferior. It resulted in no agreed upon restrictions on the research. As one participant put it, the summit was “a three day show of science explaining itself and its goals to the public and listening politely to public voices, including critical ones,” but taking no action, which made it only “successful as political theatre,” not as deliberation toward policy-making; the recommendations that came out of it were practically identical to those set out in the earlier *Science* essay (Greely 2015). If anything, the recommendations that came out of the meeting were less restrictive than those set out in the Doudna essay, with the meeting summary concluding that basic research on germline editing should proceed apace, with a mere acknowledgement that “it would be irresponsible to proceed with any clinical use of germline editing unless and until (i) the relevant safety and efficacy issues have been resolved, based on appropriate understanding and balancing of risks, potential benefits, and alternatives, and (ii) there is broad societal consensus about the appropriateness of the proposed application” (Baltimore et al. 2015b). In the Doudna essay, there was at least the hint that there might be *no* responsible uses of the technology (Baltimore et al. 2015a, 37). In the summit summary, the assumption seems to be that resolving safety and efficacy issues is just a matter of time, and important details such as the consequences for proceeding “irresponsibly,” or what counts as “broad societal consensus,” are left unstated. As another participant remarked, “the statement explicitly leaves the door unlocked and open. … In other words, the committee has kicked the can down the road” (Darnovsky 2015). For scientists to see themselves as capable of constructing, closing and locking a door, or picking up and doing something with the can of worms that is human gene editing, they have to imagine themselves as agents with some power to control or contain the technologies they create. That is a metaphorical worldview in short supply in the 2015 texts.

Burke not only introduced the concept of terministic screens and the critical practice of comparing ratios of dramatistic key terms, but also the idea of a “trained incapacity,” in which a person’s professional orientation keeps them from seeing things that are outside the boundaries of that orientation (Burke 1984). The restricted metaphoric imagination of current scientists might be partially the result of professional norms that encourage them to focus on the object of their research, subordinating themselves by avoiding personal pronouns and using the more objective-sounding passive voice. Over time, scientific communication has increasingly moved in this direction, although there is evidence that the development of this style had already stabilized by the beginning of the twentieth century (Gross et al. 2002, 63). This makes it unlikely that a perfecting of this norm explains the difference between the 1974 and 2015 texts. However, it is at least possible that in the more than forty years between these two textual artifacts, there has been an extension of this norm, with scientists now using it not only in their prototypical scientific research reports, but also in essays that are designed to promote deliberation over science policy. Such stylistic creep, from one genre to another, could reflect a troubling attitude in the current moment. When scientists remove themselves from the story not only in scientific articles but also in deliberative texts, and correspondingly animate the methods and objects of research in such discourse, it might subsequently become difficult for them to see themselves as empowered agents with a responsibility to act to control those methods and objects.

Other shifts over this forty year time period that might account for scientists adopting a reduced sense of themselves as potential agents responsible for the effective regulation of the biotechnologies they create in the laboratory are the expansion of the scientific enterprise in a global context and the rise of citizen science free from professional norms, particularly in light of $150 online CRISPR kits. As Berg put it, “the issues that challenge us today are qualitatively different: they are often entwined with economic self-interest and increasingly beset by nearly irreconcilable ethical, religious, and legal conflicts, as well as by challenges to deeply held social values” (Berg 2001, 185). A feeling of helplessness in the face of an increasingly complex and heterogeneous scientific enterprise and social world might give individual scientists little faith in their ability to constrain the technologies with which they work.

Yet another explanation for the stylistic differences that I found between these texts is one that I suggested in my earlier essay: the anthropomorphism of biotechnology might be “the dangerous upshot of the academy’s recent enchantment with a vibrant materialism coupled with its rejection of the ideology of human agency” (Ceccarelli 2018). In a postmodern scholarly scene where object-oriented ontology has become the flavor of the week, theories that invest *things* with vitality and that decenter human beings might be sapping scientists of their sense of themselves as agents with the power to defer certain types of research.

Whatever the reason for the difference between these texts from two eras, I think it is important to recognize the difference and reflect on its meaning. In the past, STS scholars have expressed concern about the way genetic engineering and synthetic biology metaphors have the potential to mechanize organisms. Carmen McLeod and Brigitte Nerlich review some of this scholarship in their introduction to this special issue (McLeod and Nerlich 2017, 9–10). The concern that I raise is different. In the texts on CRISPR-Cas9 technology that I examined in this article, it is not organisms that are being conceived as things to be taken apart and put together again like a machine. Instead, it is the gene modifying technology that is being conceived as a living thing. Conceiving of organisms as machines is problematic because it transforms what should be an ethics oriented toward living things into an ethics designed to manage things. But the reverse is true as well; conceiving of technologies as living things might be just as troubling because it takes responsibility away from the people who are using those technologies and places it in the metaphoric hands of the technologies themselves, absolving us of accountability for the acts performed.
